# HBS Ag seroclearance and seroconversion time in Patients with chronic hepatitis B Infection

**DOI:** 10.22088/cjim.11.2.205

**Published:** 2020

**Authors:** Jila Masrour-Roudsari, Mohammadreza Hasanjani-Roushan, Yousef Yahyapour, Rahim Barari-Savadkoohi, Ali Bijani, Farzin Sadeghi, Mousa Mohammadnia-Afroozi

**Affiliations:** 1 Student Research Committee, Babol University of Medical Sciences, Babol, Iran; 2 Infectious Diseases and Tropical Medicine Research Center, Health Research Institute, Babol University of Medical Sciences, Babol, Iran; 3 Social Determinants Research Center, Health Research Institute, Babol University of Medical Sciences, Babol, Iran; 4 Cellular and Molecular Biology Research Center, Health Research Institute, Babol University of Medical Sciences, Babol, Iran

**Keywords:** Chronic Hepatitis B, Seroclearance, Seroconversion, Hepatitis B Surface antigen

## Abstract

**Background:**

Hepatitis B Surface antigen (HBsAg) seroclearance and seroconversion (development of antibodies against HBsAg) can increases the survival of Chronic hepatitis B (CHB) patients. The aim of this study was to determine the percentage and timing of HBsAg seroclearance and seroconversion in patients with chronic hepatitis B infection.

**Methods:**

1026 patients with CHB infection who referred to a private clinic were included. These patients had been followed-up for an average of 15 years. The patients whose HBs Ag was cleared from the blood and remained negative until the end of follow-up were designated as HBs Ag serocleared and the patients whose HBs Ab was positive during follow-upwas designated as HBs Ag seroconverted. The time of seroclearance and seroconversion of patients was recorded. Liver function tests, alpha-fetoprotein (AFP) and Hepatitis B early antigen (HBe Ag) status were extracted from the patients’ medical records. Data were analysis with SPSS 17.

**Results:**

The duration of follow-up was from 2 to 410 months and most patients were males (58.2%).

The survival rate of HBs Ag positivity after 5, 10 and 15 years were 95.6, 89.4 and 80.7%, and 98, 93.5 and 84.9% of patients had not yet developed anti-HBs antibodies after 5, 10 and 15 years, respectively. Age, gender and taking medication had no effect on HBs Ag clearance from the blood or anti-HBs production

**Conclusion:**

The HBs Ag seroconversion is a rare occurrence, but the incidence of this may increase with time, age and drug consumption. Though there was no relationship in our patients

Hepatitis B infection is a viral disease affecting a large population worldwide and a major global health problem because of its important role in human mortality (1, 2). It is one of the main causes of cirrhosis and hepatocellular carcinoma (HCC) and as an important cause of death in patients (3, 4). Due to the interaction between virus and host, hepatitis B virus infection presents itself in a variety of clinical manifestations including acute hepatitis chronic hepatitis hepatic cirrhosis, , and HCC (5). In acute hepatitis, an adequate and timely immune response can lead to the clearance of the virus, resulting from the activity of different types of T cells. However a deficient T cell response can lead to chronic viral infection and progression to cirrhosis and HCC. Several studies have shown that different phases of the disease are caused by the virus and altered host immune response. 

Interaction between the virus, liver cells, immune system and antiviral therapy sometimes causes the virus gene to mutate, leading to the virus escaping from the immune system and drug resistance (6-8). In the normal course of chronic hepatitis B infection, hepatitis B surface antigens (HBs Ag) may be cleared from the patient's blood. In this phase of the disease, antibodies to the hepatitis B core antigen (HBcAg) are found in the blood, and antibody against HBsAg (Anti-HBs) may or may not be found. HBs Ag clearance can prevent progression of disease to cirrhosis or HCC and increase the survival of affected patients. (6, 9, 10). HBs Ag seroclearance is a rare event in chronic hepatitis B. The incidence of HBs Ag seroclearance in patients affected with chronic hepatitis early in life is estimated to be about 0.1 to 0.8% per year, and be 0.4-2% in patients affected in adulthood. Although it is believed that HBs Ag seroclearance in patients with chronic hepatitis B is a good sign of inprogression to HCC, the patients may develop cirrhosis and HCC despite HBs Ag clearance from blood (4, 9). Several studies have indicated that HBs Ag seroclearance can be a sign of effective treatment for patients with hepatitis B infection (4, 11-13). Despite this fact the spontaneous clearance of HBs Ag in untreated patients is rare, it may occur in the normal course of the disease (4). Factors such as age, gender, drug consumption and virus DNA level are effective at the time of seroclearance and seroconversion (14). Therefore, the aim of this study was to determine the percentage and timing of HBs Ag seroclearance and HBs Ag seroconversion in 1026 patients with chronic hepatitis B, followed-up for an average of 15 years and investigate some of its contributing factors.

## Methods

In a historical cohort study, from 2004 to 2019 in Babol, 1026 patients who referred to a private clinic with positive HBs Ag for 6 months were included. The data was extracted from the patients’ medical records. These patients had been followed-up for an average of 15 years. The HBs Ag, HBs Ab, liver enzymes and alpha-fetoprotein (AFP) HBeAg of patients were measured every 6 months.

The patients whose HBs Ag was cleared from the blood and remained negative until the end of follow-up were designated as HBs Ag serocleared and the patients whose HBs Ab was positive during follow-up was designated as HBs Ag seroconverted. The HBs Ab>10 was considered as positive. The time of seroclearance and seroconversion of patients and also the last alanine aminotransferase (ALT) and aspartate aminotransferase (AST) was recorded. The range for normal AST and ALT are considered 5 to 50 units per liter. Patients with concomitant hepatitis C, hepatitis D, cirrhosis or HCC were excluded from the current study. 

**Statistical analysis:** Data were analyzed using SPSS 17. Descriptive data were reported in frequency, mean and standard deviation. The Kaplan-Meier method and Cox regression model were used for survival analysis. Crude and adjusted hazard ratios (HR) with 95% confidence intervals (CI) were reported, and p<0.05 was considered as significant level.

## Results

 Totally, 1026 HBs Ag^+^ patients whose HBs Ag remained positive for at least 6 months were retrospectively reviewed. The demographic characteristics of these patients are summarized in table 1.

**Table 1:** Baseline characteristics of chronic hepatitis B patients Characteristics

**Age of initial visit (mean±SD)**	**30.82±11.27**
Sex, N (%)	
Male female	597 (58.2) 429 (41.8)
Drug consumption, N (%)	
Yes No	38 (3.7) 988 (96.3)
Age (Year), N (%)	
<30 ≥30	594 (57.9) 432 (42.1)
Liver Function Tests (Mean±SD) (IU/L)	
ALT AST	33.5±15.1 37.3±16.2
HBs Ag – During follow up, N (%)	93 (9.1)
HBs Ab + During follow up, N (%)	57 (5.6)

**Figure 1:**
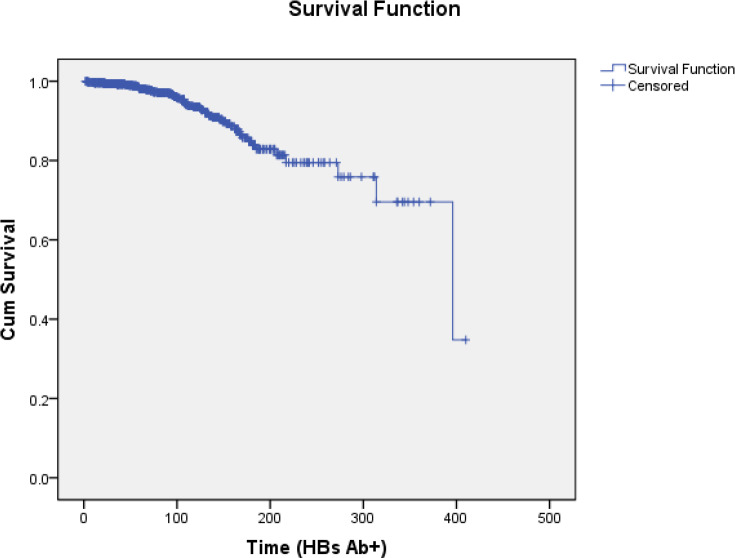
Cumulative survival rate of HBs Ag positivity during follow-up

The age of the patients ranged from 4 to 83 years with the mean age of 30.82±11.27 years. The duration of follow-up was from 2 to 410 months and most patients were males (58.2%). All patients were Hepatitis B early-antigen (HBe Ag) negative and their ALT and AFP levels were normal in the last test recorded at follow-up. Of the 93 patients with HBs Ag negative, 52 were antibody positive, and antibodies were formed in the blood of 5 patients despite the presence of positive antigen. Out of the 38 patients who took the drug, 1 case was serocleared and 1 patient was seroconverted. The survival rate of HBs Ag positivity after 5, 10 and 15 years were 95.6, 89.4 and 80.7%, respectively (table 2, figure 1).

**Table 2:** Survival rate of HBs Ag positivity during follow-up

**Follow-up duration (year)**	**Survival ±SE**
5	95.6±0.7
10	89.4±1.4
15	80.7±2.5
20	73.3±3.7
25	66.8±5.5
30	43.8±11.7

**Figure 2:**
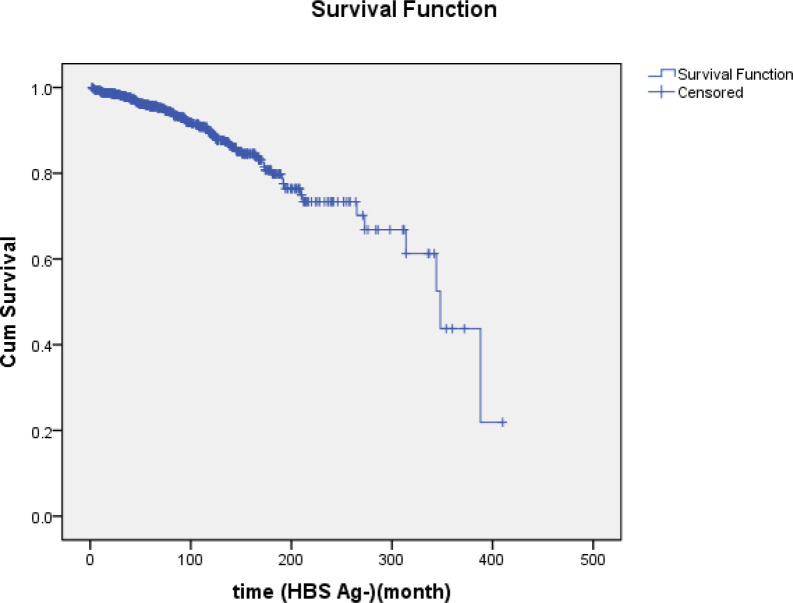
Cumulative survival rate of HBs Ab negativity in CHB patients during follow-up

Seroconversion was not developed in 98, 93.5 and 84.9% of patients after 5, 10 and 15 years, respectively (table 3, figure 2).

**Table 3:** Survival rate of HBs Ab negativity in CHB patients during follow-up

**Follow-up duration (year)**	**Survival ±SE**
5	98±0.5
10	93.5±1.1
15	84.9±2.4
20	79.5±3.5
25	75.9±4.9
30	69.6±7.5

Moreover, , Cox regression analysis indicated that age, gender and taking medication had no effect on HBs Ag seroclearance and seroconversion (tables 4 and 5).

**Table 4:** Multivariable Cox regression model of the factors affecting HBs Ag negativity in patients with CHB

**Variables**	**HR (95% CI)**	**p-value**
Sex	1.27 (0.828-1.972)	0.269
Age >30	1.261 (0.833-1.911)	0.273
Drug	0.244 (0.34-1.256)	0.161

**Table 5:** Multivariable Cox regression model of factors affecting HBs Ab positivity in patients with CHB

**Variables**	**HR (95% CI)**	**p-value**
Sex	1.431 (0.826-2.477)	0.201
Age >30	1.329 (0.782-2.259)	0.293
Drug	0.764 (0.186-3.142)	0.709

## Discussion

In the present study cumulative incidence of HBs Ag seroclearance, HBs Ab seroconversion and possible factors affecting patients outcome were investigated. Totally, HBsAg seroclearnce and seroconversion was observed in 9.1%and 5.6% of patients respectively. HBsAg seroclearance and seroconversion are important events in the course of hepatitis B infection that can be good prognostic factors in disease outcome. The incidence rate of HBsAg seroclearnce is very low (1.6-1.8% per year), representing a rare event in the natural history of HBV infection..However, some studies have suggested that HBsAg seroclearance is more likely to occur due to aging and antiviral therapy in chronic hepatitis B patients (3, 9, 15). The results of current study were not in line with several investigations which considered age and antiviral therapy as factors that affect HBsAg seroclearance and seroconversion.

Some risk factors for progression of chronic hepatitis B to seroclearance have been reported. Older age is a strong predictor of progression, but this is more likely due longer duration of disease rather than an independent risk factor. Age at entry has been identified as factor associated with increased HBsAg seroclearance (16-18).

Another commonly associated risk factor is male sex although the exact mechanism is not clear (19). Spontaneous hepatitis B surface antigen (HBsAg) seroclearance is associated with a good prognosis in occurring during the inactive phase of chronic hepatitis B virus (HBV) infection Patients. is associated with excellent prognosis. HBsAg levels declined decreased to less than 200 and less than100 IU/mL in 55% and 37% of the patients, respectively, at 5 years before HBsAg seroclearance. The proportion part of patients reaching these levels of HBsAg increased to 100% and 98% at 1 year, respectively, and 81% and 64% at 3 years, respectively, before HBsAg seroclearance. 

Itaru Ozeki et al. showed that during the mean follow-up period of 31 months, the HBsAg seroclearance rates determined by high-sensitivity HBsAg assays in discrepant cases were 20.8% at 1 year, 40.6% at 2, 51.3% at 3, 64.3% at 4, and 74.5% at 5 years, respectively. The cumulative anti-HBs positivity rates in discrepant cases were 12.7% at baseline, 17.2% at 1 year, and 38.8% at 3 years (15), which were significantly higher than those in the present study. Of course, the number of patients in their study was smaller than ours.

In a study, Yen et al. illustrated that there was a significant relationship between HBs seroclearance with male gender and age over 60 years in patients. In their study, after treatment with interferon, 53 (26.4%) of 201 patients were HBs-Ag positive and among these patients, 15 patients developed anti HBs antibody. Moreover, 9.8 and 47.07% of patients were serocleared in 5 and 10 years, respectively, which is inconsistent with our study, indicating that treatment can be effective in seroclearing the patients (20). However, in our study, the duration and type of antiviral therapy was different. Like the present study, Fung et al. found that the HBs Ag seroclearance was 1.1%, 3.3%, 13.5% and 23.4% in 5, 10, 20 and 30 years after patients undergone HBe Ag seroclearance, respectively. In addition, in their study, the age had no effect on seroclearace, but patients who needed medication were serocleared later than those without medication. This may be because the patients who did not require medication were probably better in terms of status, number of viruses and blood biochemical factors.

It was suggested that patients who were HBs Ag serocleared or HBs Ag seroconverted were less likely to develop HCC during follow-up. As the probability of viral rebound is negligible through HBs Ag seroclearance; therefore, it can be an appropriate endpoint for physicians to terminate (10).

Zu et al. have demonstrated that if a person becomes infected with hepatitis B at an early age, it is more likely to be serocleared and seroconverted, which may be due to vaccination against hepatitis B at this age. Their study also illustrated that drug consumption increased the percentage of HBs Ag clearance from blood and antibody production, which disagrees with our results so this difference appears to be due to our retrospective study design and lack of monitoring of drug consumption (21).

The study of Faisal et al. has indicated that higher the age of hepatitis in addition to the lower percentage of serocleared and seroconverted patients, the more likely the liver to develop fibrosis because in the older age, tissue regeneration is lower. Although the drug can be effective in HBs Ag seroconversion of patients, the type of drug is also effective. For example, the nocleosid analogs are unable to cure infection because they cannot remove cccDNA of the virus from the nucleus of hepatocytes (22). In our study, the drug had no significant effect on the course of the disease, which might be due to the type of drug being used.

In the present study, there were some limitations. The first limitation was the retrospective study design; thus, the prospective studies are needed to follow-up patients more closely. Since HBs Ag seroclearance and HBs Ag seroconversion are rare, the low number of patients as well as patients taking medication was the second limitation of the study.

Despite the limitations of the study, the results have shown that during hepatitis B, the HBs Ag may be cleared in some patients, and even in some cases, the anti-HBs Ag antibodies may be formed. This is a rare occurrence, but it seems that the incidence of this may increase with time, age and drug consumption. Though there was no relationship between seroclearance with age and drug consumption in patients, further prospective studies with larger sample size may yield better results.
